# Preparation, Characterization, and Biocompatibility Assessment of Polymer-Ceramic Composites Loaded with *Salvia officinalis* Extract

**DOI:** 10.3390/ma14206000

**Published:** 2021-10-12

**Authors:** Dagmara Słota, Wioletta Florkiewicz, Karina Piętak, Aleksandra Szwed, Marcin Włodarczyk, Małgorzata Siwińska, Karolina Rudnicka, Agnieszka Sobczak-Kupiec

**Affiliations:** 1 Department of Materials Science, Faculty of Materials Engineering and Physics, Cracow University of Technology, 37 Jana Pawła II Av., 31-864 Krakow, Poland; wioletta.florkiewicz@pk.edu.pl (W.F.); karina.pietak6@gmail.com (K.P.); agnieszka.sobczak-kupiec@pk.edu.pl (A.S.-K.); 2 Department of Immunology and Infectious Biology, Faculty of Biology and Environmental Protection, University of Lodz, 12/16 Banacha, 90-237 Lodz, Poland; aleksandra.szwed@biol.uni.lodz.pl (A.S.); marcin.wlodarczyk@biol.uni.lodz.pl (M.W.); karolina.rudnicka@biol.uni.lodz.pl (K.R.); 3 Department of Biology of Bacteria, Faculty of Biology and Environmental Protection, University of Lodz, 12/16 Banacha, 90-237 Lodz, Poland; malgorzata.siwinska@biol.uni.lodz.pl

**Keywords:** common sage, *Salvia officinalis*, hydroxyapatite, ceramics, polyvinylpyrrolidone, composite

## Abstract

In the present work, hydroxyapatite-polymer materials were developed. The preparation, as well as characterization of the ceramic-polymer composites based on polyvinylpyrrolidone, sodium alginate, and gelatin were described. The system was enriched with the addition of common sage extract (*Salvia officinalis*). The antioxidant potential of sage aqueous extract and total polyphenol content was determined. The antioxidant capacity and total phenolic content of extract were equal to 86.06 ± 0.49% and 16.21 ± 0.58 mg gallic acid equivalents per gram of dry weight, respectively. Incubation studies in selected biological liquids were carried out to determine the biomineralization capacity on the surface of the composites and to examine the kinetics of release of the active substances from within the material. As a result of the incubation, a gradual release of the extract over time from the polymer matrix was observed; moreover, the appearance of new apatite layers on the composite surface was recorded as early as after 14 days, which was also confirmed by energy-dispersive X-ray spectroscopy (EDS) microanalysis. The composites were analyzed with Fourier transform infrared spectroscopy (FTIR) spectroscopy, and the morphology was recorded by scanning electron microscope (SEM) imaging. The in vitro biological studies allowed their cytotoxic effect on the reference L929 fibroblasts to be excluded. Further analysis of the biomaterials showed that enrichment with polyphenols does not support the adhesion of L929 cells to the surface of the material. However, the addition of these natural components stimulates human monocytes that constitute the first step of tissue regeneration.

## 1. Introduction

Most organ and tissue damage resulting from congenital defects, trauma or chronic diseases is treated surgically, or pharmacologically. This includes the use of appropriate medications or, in more severe cases, organ transplants [1,2]. However, for some time now, great emphasis has been placed on intelligent, bioactive biomaterials, which offer great potential for regenerative medicine, by, among other things, stimulating the surrounding tissues or delivering active components such as drugs or biomolecules [3,4]. Such biomedical materials are widely used in orthopedics as implants, dressings and bone defect fillers and should have ideal biocompatibility, biodegradability and bioactivity [5,6]. In the case of bone tissue, the ability for osteoconduction as well as osteoinduction is also extremely important [7]. For bone tissue regeneration, all types of materials available are used, medical polymers, metals like titanium and its alloys but mainly it is bioceramics whose chemical composition is highly similar to that of natural bone [8,9].

Bone is a heterogeneous composite material consisting of both organic and inorganic components [10]. The organic part, represented mainly by type I collagen fibrils, constitutes about 25–30% of the net weight of bone, about 60–70% is the mineral phase, and the remaining 5–10% is water [11,12]. The organic part provides flexibility and elasticity to the tissue, while the mineral part contributes to the stiffness and load bearing capacity of bone. The main component of the inorganic phase is the calcium phosphate ceramic, hydroxyapatite (HA) Ca_10_(PO_4_)_6_(OH)_2_ [13,14]. Synthetic HA is widely used in medicine and dentistry in powder form for filling cavities or as a covering material for metallic implants [15]. It shows high biocompatibility as well as the ability to promote the infiltration of bone, bone marrow and blood vascular cells [16,17]. It is worth emphasizing that HA does not stimulate bone formation in the traditional sense, however, it directs its growth, which is referred to as osteoconductive action [18,19]. The limiting factors for HA applications are weakness in shear and tension as well as brittleness [20]. However, due to the ease of combining ceramics with other materials, especially polymers, these disadvantages can be overcome by suspending the ceramics in a polymer matrix, leading to a composite material. This results in a biomaterial with improved mechanical properties without losing the bioactive nature of HA [21,22].

In the present work, the polymer phase consisted of polyvinylpyrrolidone (PVP). It is an essential, water-soluble polymer approved by the U.S. Food and Drug Administration (FDA) as safe for body contact [23,24]. It shows excellent physiological compatibility, no toxicity and good adhesion [25,26]. For many years, it has been used in the medical industry as, for example, a component of contact lenses, a binder in pharmaceutical tablets and as a filler and binder in cosmetic products [27,28,29].

Common sage (*Salvia officinalis*), also called garden sage, kitchen sage or Dalmatian sage, is a plant of the *Lamiaceae* family, widely cultivated in the Mediterranean basin [30,31]. Besides its use as a popular spice, sage has been documented as a general tonic or medicine in consideration of its purported widespread medicinal properties [32]. The etymology of the Latin word *Salvia* is derived from the phrase salvare meaning “to cure” and salvere meaning “to be healthy”. The second part, *officinalis*, which is a species name, refers to its common medicinal use [33]. In traditional medicine, it is used to treat various conditions, including diarrhea, gout, inflammation, rheumatism, ulcers, epilepsy and hyperglycemia [34]. According to the Pharmacopoeia, the pharmaceutical raw material of clary sage is the leaves and leafy shoot tips as these parts are the most rich in active substances [35]. Caffeic acid derivatives and widely distributed flavonoids, occurring mainly in the form of flavones (e.g., apigenin, luteolin and their respective 6-hydroxylated derivatives), flavonols and their glycosides (e.g., kaempferol and quercetin), play a significant role in sage activity [36,37]. By the presence of the mentioned components, the plant is attributed with antioxidant, antimicrobial, antiproliferative and anticarcinogenic properties [38]. Its ability to inhibit bone tissue resorption seems to be of particular interest [39]. Therefore, the combination of the osteoconductive properties of hydroxyapatite with the biological activity of sage may enhance bone regeneration.

The aim of this study was to develop a ceramic-polymer composite modified with plant extract using biocompatible PVP, bioactive HA showing osteoconductive properties and common sage extract to increase the biological value of the biomaterial. According to our best knowledge, any measurement methodology aimed at determining the structure–property relationships in such ceramic-polymer composites modified with common sage extract has not yet been presented.

## 2. Materials and Methods

### 2.1. Reagents

Common sage (*Salvia officinalis*) dried leaves were purchased from Lord Nelson (Tarnowo Podgórne, Poland). All reagents used for HA synthesis, i.e., the sodium phosphate dibasic (Na_2_HPO_4_), calcium acetate monohydrate (Ca(CH_3_CO_2_)_2_·H_2_O), ammonium phosphate monobasic and ammonia water (NH_4_OH, 25%) as well as sodium alginate (SA), polyvinylpyrrolidone (PVP), poly(ethylene glycol) diacrylate average Mn 700 (PEGDA), gelatin (GE), 2-hydroxy-2-methylpropiophenone 97%, 2,2-Diphenyl-1-picrylhydrazyl (DPPH), triethanolamine ≥ 99.0% (TEA), gallic acid and edetate disodium were obtained from Sigma-Aldrich (Darmstadt, Germany). Urea was purchased from Stanlab (Lublin, Poland) and phosphate buffered saline (PBS) from Oxoid (Basingstoke, UK). The bismuth(III) nitrate pentahydrate (Bi(NO_3_)_3_), sodium carbonate (Na_2_CO_3_), potassium chloride (KOH), sodium chloride (NaCl), nitric acid (HNO_3_), hydrochloric acid (HCl), ethanol 96%, calcium chloride dihydrate (CaCl_2_·2H_2_O) and sodium sulfide nonahydrate (Na_2_S · 9H_2_O) were obtained from Chempur (Piekary Śląskie, Poland).

### 2.2. Preparation of Hydroxyapatite

Hydroxyapatite (HA) was synthesized by wet precipitation method performed at boiling temperature. Distilled water and a defined volume of Na_2_HPO_4_ (0.32 mol/L) were poured into a three-necked flask. Using 25% ammonia water, the pH of the system was brought to a level of 11. Once the entire system was brought to a boil, (CH_3_COO)_2_Ca (0.128 mol/L) was dropped in at a rate of 1 drop/sec. After completion of the synthesis, the HA suspension was cooled down and set aside for 24 h. After this time, the precipitate was washed thoroughly with distilled water, brought to neutral pH and dried [22].

### 2.3. X-ray Diffraction Analysis

To visualize the structure of HA, X-ray diffraction analysis was performed using a Malvern Panalytical Aeris X-ray diffractometer with PIXcel1D-Medipix3 detector (Malvern, UK). The measurement was carried out at a step size of 0.0027° 2θ in a 2θ range of 25–55° with time per step 340.425 s. 

### 2.4. Preparation of Common Sage Ectract 

The process of aqueous extraction of *S. officinalis* was carried out in a Soxhlet apparatus. For this purpose, a sample of dried sage was placed in an extraction thimble and distilled water was poured into a round-bottomed flask. The system was brought to a boil and the process was carried out for 12 h.

### 2.5. Preparation of Composites

A 15% solution of PVP, a 1% solution of GE and a 1% solution of SA were prepared to obtain mixtures for the production of composites. Appropriate amounts of such prepared solutions as well as HA powder were used to obtain the blends used in the preparation of composites. 2-hydroxy-2-methylpropion and PEGDA Mn 700 were used as a photoinitiator and crosslinking agent, respectively. The materials containing common sage extract were prepared by dissolving an appropriate amount of PVP in polyphenol solution. The detailed compositions of the composites are presented in Table 1.

The components were mixed thoroughly and poured on a Petri dish. The photocrosslinking was performed using EMITA VP-60 lamp (Łódź, Poland) (λ = 320 nm, 180 W) for 4 min. 

### 2.6. Fourier-Transform Infrared Spectroscopy Analysis

To identify individual functional groups of the HA, PVP and sage samples, as well as to perform composite analysis before and after incubation period, Fourier transform infrared spectroscopy (FT-IR) was used. Analysis was performed with a Thermo Scientific Nicolet iS5 FT-IR spectrometer equipped with an iD7 ATR (Loughborough, UK) accessory operating at room conditions in the range of 4000−400 cm^−1^ (32 scans at 4.0 cm^−1^ resolution).

### 2.7. Incubation in Vitro

#### 2.7.1. pH-Metric Analysis

To evaluate the bioactivity of the obtained sage composite materials in vitro, a pH-metric study was performed. The purpose of this research was to confirm the interactions occurring between the sample and the incubation fluids. The molecules and ions contained in the fluids, interfering with the biomaterial, cause a change in pH value. Discs with mass of about 1 g were placed in simulated biological fluids, i.e., PBS, artificial saliva, and Ringer’s fluid (60 mL). The biomaterials were incubated in POL-EKO incubator, model ST 5 B SMART (Wodzisław Śląski, Poland) at 37 °C; for 14 days. The pH values were measured using pH-meter Elmetron CX-701 (Zabrze, Poland).

#### 2.7.2. Determination of Sorption Capacity of Biomaterials

The composition of hydrogel materials may affect not only their structure but also the materials’ swelling ability. To investigate this composition−swelling capacity relationship, a swelling kinetic study was proposed. The sorption capacity of materials was measured for disks with mass of about 1 g. The aim of the study was to determine the amount of fluid that materials can absorb in a given time. For this purpose, the samples were placed in sterile containers filled with distilled water (60 mL) at 36.6 °C. After 15 min, the sample was removed, excess liquid was collected with a filter paper and specimens were weighed. The measurement was repeated for all samples analogously at 15 min, 30 min, 1 h, 2 h, 24 h, 7 days, and 14 days. The swelling ratio (*S_w_*) was calculated using the following Equation (1):

(1) $$S_{w}=\frac{W_{t}-W_{0}}{W_{t}}\cdot 100\%$$

where *W_t_* is the weight of the swollen hydrogel sample and *W_0_* is the initial sample weigh.

The kinetic of swelling of the materials was investigated by Voigt-based viscoelastic model (Equation (2)):

(2) $$S_{t}=S_{e}\left[1-e^{-\frac{t}{\tau }}\right]$$

where *St* is swelling at time t, *Se* is equilibrium swelling, *t* is time for swelling *St*, and *τ* means “rate parameter”, defined as the time taken for the sample to reach 0.63 of its total swelling capacity. To determine *Se* and *τ* results of the swelling capacity measurements were fitted to Equation (2) with the Origin software [40]. 

### 2.8. Determination of Total Polyphenol Content

The Folin−Ciocâlteu colorimetric assay was performed to determine the total polyphenol content (TPC) of the tested extracts. A supersaturated solution of Na_2_CO_3_ and a gallic acid solution of 5 mg/mL were prepared. The solutions were obtained by dilution of a working solution of gallic acid (5 mg·mL^−1^) to the concentrations: 0.05; 0.15; 0.25; 0.35; 0.5 mg/mL to form a calibration curve. Next, 20 μL of calibration standards, 1.6 mL of distilled water, and 100 μL of Folin−Ciocâlteu reagent were added to the cuvette. After approximately 3 min, 300 μL of saturated Na_2_CO_3_ solution was added. The prepared samples were thoroughly pipetted to evenly mix, and placed in a thermostat for 30 min at 40 ℃. 

Test samples of the resulting extracts were prepared in an identical manner, replacing the gallic acid solution with 20 μL of extract of common sage, which had been diluted three times. Measurements were carried out at 765 nm against a blank, which was a sample without gallic acid. A Thermo Scientific, Genesys 180 UV−Vis spectrophotometer (Loughborough, UK) was used for the measurements.

### 2.9. Determination of Antioxidation by DPPH Method

To investigate the antioxidant efficacy of the selected extracts, their ability to inactivate free radicals was examined. The antioxidant activity was determined using 2,2-Diphenyl-1-picrylhydrazyl (DPPH). A solution of DPPH was prepared by dissolving 19.71 mg of this compound in 100 mL of 96% ethanol. The resulting solution was then diluted to an absorbance value of about 0.9. The control sample *A*_0_ was prepared by adding 2 mL of the prepared DPPH solution and 60 μL of 96% ethanol. Absorbance was measured on a UV−Vis spectrophotometer at 517 nm. Samples with extracts of clary common sage were measured in an identical manner, replacing 60 μL of ethanol with the selected extract. Absorbance was measured after approximately 10 min at room temperature at 517 nm. Three replicates were made for each sample and averaged (*A*). The antioxidant capacity of the test extract was calculated from the formula (3) [41].

(3) $$\%\;inhibition=\frac{A_{0}-A}{A_{0}}\cdot 100$$

### 2.10. Determination of Release Kinetics of Polyphenols

In order to study the amount of release of the active ingredient from the obtained composites with common sage, an Electrolab EDT-08lx (Mumbai, India) release water bath was used. The composite samples: 17.2 and 25.2 were placed in the release benches with stirring. Distilled water was then added and stirring was turned on at 36.6 °C. After the specified time, 1 mL of solution was taken from each station, and 1 mL of distilled water was added to release benches. Samples were taken at intervals of: 15 min, 30 min, 90 min, 1 day, 2 days, 3 days, 4 days, 7 days, 10 days, and 14 days. The obtained samples were subjected to tests for determination of total polyphenol content (TPC) by the Folin−Ciocâlteu method [41].

### 2.11. Morphology Analysis

Surface morphology studies of the samples were performed using a Jeol 5510LV Scanning Electron Microscope (SEM) with an EDS IXRF System detector (Freising, Germany). SEM analysis was performed for the sake of comparing the surface of the composites before and after incubation period in artificial biological fluids to detect any changes as well as to record new apatite layers appearing on the surface of the samples. 

Before the SEM measurement, the samples were freeze-dried and coated with nano-layer of gold.

### 2.12. Cytocompatibility of the Composites

#### 2.12.1. Sample Preparation for Biological Studies 

Prior to the biological evaluation, all composites were sterilized by gamma-irradiation (35 kGy gamma rays; ^60^Co source) at the Institute of Applied Radiation Chemistry, Technical University in Lodz (Lodz, Poland). The effectiveness of sterilization was confirmed by conventional microbiological techniques eri. Briefly, each type of sample was incubated in 5 mL of PBS/Tween buffer at room temperature (15 min./shaking) and 0.1 mL of the resulting liquid was transferred onto solid microbiological media for bacteria or yeast (Sabouraud agar): and incubated at 37 °C for 24 h or 5 days, respectively.

For cytotoxicity (Section 2.12.3) and proinflammatory (Section 2.12.4) assay, composites were cut into pieces corresponding to one-tenth of the well surface area, as recommended by ISO 10993-5:2009 (Biological evaluation of medical devices—Part 5: Tests for in vitro cytotoxicity). To evaluate the composites in regard to cell expansion and adhesion by confocal microscopy imaging (Section 2.12.5) samples were cut into 7 mm diameter discs.

#### 2.12.2. Cell Culture Conditions

The L929 (CCL-1) mouse skin fibroblasts recommended by ISO standards for biological evaluation of biomaterials with medical applications were obtained from the American Type Culture Collection (ATCC, Manassas, VA, USA). Prior to experiments, fibroblasts were cultured in Roswell Park Memorial Institute (RPMI)-1640 medium supplemented with 10% heat-inactivated fetal bovine serum (FBS; HyClone Cytiva, Marlborough, MA, USA), penicillin (100U/mL), and streptomycin (100 µg/mL) (Sigma-Aldrich, Darmstadt, Germany) at a temperature of 37 °C, in a humidified air atmosphere containing 5% CO_2_. To ensure that the cells formed confluent and homogeneous monolayers, the cell culture morphology was monitored using an inverted microscope (Motic AE2000 Xiamen, China). The confluent (80–90%) cell monolayers were periodically subcultured using a 0.5% trypsin-0.5 mM ethylenediaminetetraacetic acid tetrasodium salt (EDTA) solution (Gibco, Thermo Fisher Scientific, Waltham, MA, USA).

The THP1-Blue NF-κB human monocytes, carrying NF-κB-inducible SEAP (secreted embryonic alkaline phosphatase) reporter construct, were obtained from InvivoGen (San Diego, CA, USA). Monocytes were cultured in RPMI 1640 medium supplemented with 10% heat-inactivated FBS, 2 mM L-glutamine, 100 U/mL penicillin, 100 μg/mL streptomycin, 25 mM 4-(2-hydroxyethyl)-1-piperazineethanesulfonic acid (HEPES), 100 μg/mL normocin and 10 μg/mL blastocydin. The cell culture was conducted at 37 °C in 5% CO_2_ atmosphere and 90% humidity.

Prior to experiments, cell viability was established using a trypan blue exclusion assay. The cell suspensions with viability exceeding 90% were used in cytocompatibility experiments.

#### 2.12.3. Direct Contact Cytotoxicity Assay

The metabolic activity of cells exposed to tested biomaterials was evaluated by 3-(4,5-dimethylthiazol-2-yl)-2,5-diphenyltetrazolium bromide (MTT) reduction assay as described previously [42]. Briefly, L929 fibroblasts were introduced into 96-well cell culture plate (Nunclon Delta Surface, Nunc, Rochester, NY, USA) at a density of 2 × 10^4^ cells per well and incubated overnight. Next, the previously prepared (Section 2.12.1) composites were added to cell monolayer (in six replicates). After overnight incubation, 20 µL of MTT (Sigma-Aldrich, Darmstadt, Germany) was added and after 4 h incubation the supernatants were removed and replaced with 200 µL of DMSO (dimethyl sulfoxide). Absorbance was determined calorimetrically at 570 nm with a MultiskanEX reader (Thermo Scientific, Waltham, MA, USA). The cell cultures in medium without the tested materials were used as a positive control of viability and cell cultures treated with 2% hydrogen peroxide served as a negative control of viability. The assay was performed in six replicates and three technical repeats. 

#### 2.12.4. Monocyte Activation Assay

To quantify the NF-κB induction activated in monocytes by obtained composites THP-1Blue NF-κB reporter cell line was used. The assay was performed as described previously [42]. Briefly, THP1-Blue NF-κB monocytes were adjusted to 5 × 10^5^ cells/mL and 200 µL of cell suspension was transferred to each well. Next, previously prepared (see Section 2.12.1) composites were added to selected wells (in six replicates) and after 24-h incubation at 37 °C in 5% CO_2_ atmosphere, the concentration of alkaline phosphatase in supernatants was measured using the QUANTI-Blue reagent (InvivoGen, San Diego, CA, USA) according to the manufacturer’s instructions. The quantification of media color change was performed with a Multiskan EX reader (Thermo Fisher Scientific, Waltham, MA, USA) at 650 nm. The monocytes activated with phorbol 12-myristate 13-acetate (PMA) at 100 ng/mL were used as positive control of NF-κB activation, whereas untreated cells were used as a negative control of activation. The assay was performed in six replicates and three technical repeats.

#### 2.12.5. Composite Colonization by Cells

To fully address the question whether obtained composites facilitate cell adhesion and expansion on their surface, we have visualized cells colonizing each material after 5 days culture. Previously prepared composites (Section 2.12.1) were placed in an individual well of the nonadherent Nunclon Delta Surface 24-well culture plate (Nunc, Thermo Fisher Scientific, Waltham, MA, USA) in three replicates each. Then 20 µL of L929 cell suspension (2 × 10^5^ cells) was spotted at the center of each biomaterial and the composites were incubated for 2 h in a humidified 5% CO^2^ atmosphere at 37 °C. After initial adhesion, the wells were filled with 1 mL of fresh medium and incubated for 5 days. Following incubation, the culture medium was removed and composites were washed with phosphate buffered saline (PBS). Next, the cells were fixed with 3.7% paraformaldehyde (Sigma-Aldrich, Saint Louis, MO, USA) for 20 min at room temperature, followed by permeabilization (15 min, 0.1% Triton X-100 in PBS). The nuclei were stained with 300 nM 2-(4-amidinophenyl)-1H-indole-6-carboxamidine (DAPI) and actin filaments with phalloidin conjugated with iFluor 594 (Cayman Chemical, Ann Arbor, MI, USA) diluted 1:1000 in PBS containing 1% BSA. The confocal laser scanning macroscopy platform TCS LSI (Leica Microsystems, Frankfurt, Germany) with the objective 5×/0.50 LWD (Leica Microsystems) was used for microscopic imaging. Samples were imaged with the following wavelength values of excitation and emission: 405 and 430–480 nm for DAPI, 590 and 615–630 nm for iFluor 594 conjugated antibody. Leica Application Suite X (LAS X; Leica Microsystems) was used for cell imaging.

#### 2.12.6. Statistical Analysis

The results were subjected to statistical analysis with the use of SigmaPlot 12.0 software (San Jose, CA, USA). In the case of normal data distribution, the t-test was used and Mann−Whitney rank sum test was used when the distribution was recognized as normal. To assess the significance of differences between particular composites, ANOVA test was conducted and for significant comparisons further analysis using the Luke’s method was employed. The differences were considered significant when a *p*-value < 0.05.

## 3. Results

### 3.1. X-ray Diffraction Analysis

**Figure 1:**
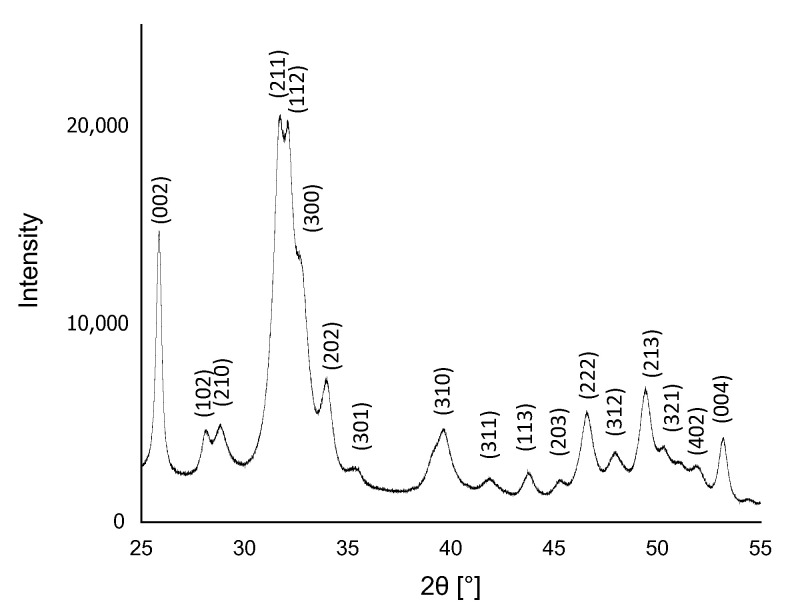
XRD spectrum of HA obtained by wet precipitation method.

The result of XRD analysis of HA powder is presented in Figure 1. The analysis showed that the obtained material was phase pure, and the only phase identified in the X-rays was HA. The result appeared to be consistent with the phases listed in the ICDD database File Card No. 01-080-7085, and the XRD reflections were assigned to the hexagonal structure (P63/m space group) of HA with the lattice parameters a = 9.4172 Å and c = 6.8799 Å [43]. 

### 3.2. Fourier−Transform Infrared Spectroscopy Analysis

**Figure 2:**
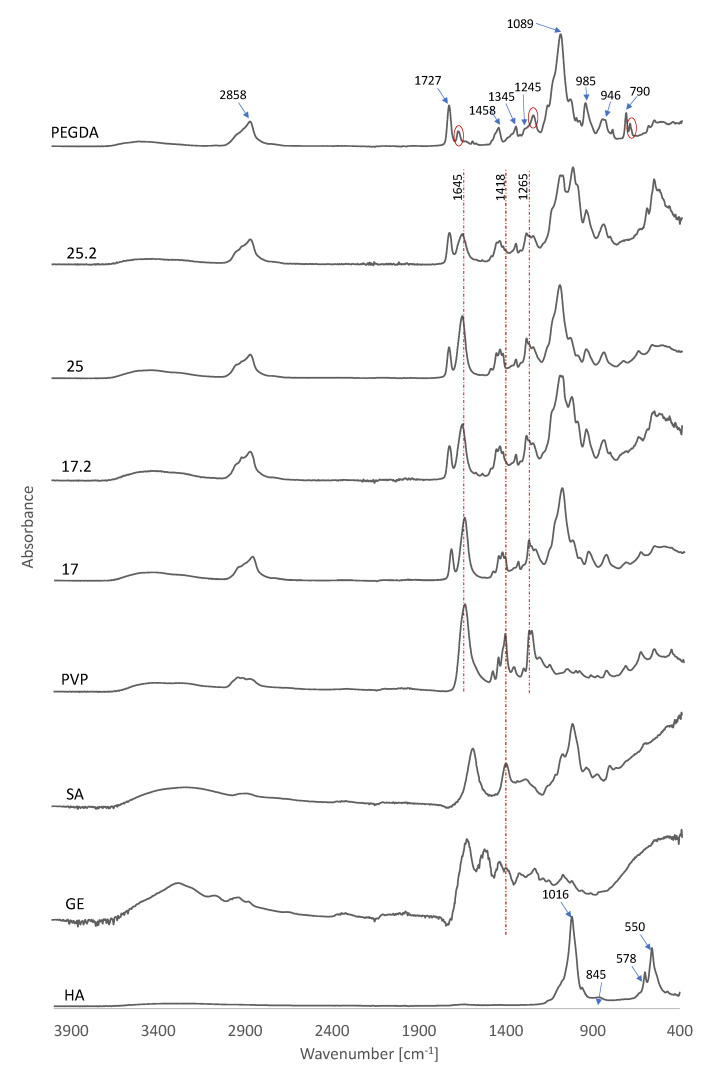
FTIR spectra of PEGDA, PVP, GE, SA, and composites.

Figure 2 presents the absorption spectra for pure components as well as composites. The absorption band of the PEGDA sample at 2858 cm^−1^ was attributed to the C-H stretching vibration, while 790 cm^−1^ represented the C-H bending mode. These C-H stretching vibrations are also clearly visible in composites and PVP. The peak at 1727 cm^−1^ was observed in association with the C=O stretching frequency [44]. This peak was also clearly visible on the spectrum of samples 17, 17.2, 25, and 25.2. The peak observed at 1458 cm^−1^ is attributed to symmetric bending of the CH_2_ group. In addition, the bands located at 1345 cm^−1^ and 1245 cm^−1^ were marked and were consistent with asymmetric C-O bending, while peaks at 1089 cm^−1^, 985 cm^−1^, and 946 cm^−1^ represented C-O-C stretching. The absorption bands indicated by the red dashed line were attributed to symmetric C=C stretching [45].

Spectral analysis of HA revealed the presence of phosphate groups as evidenced by distinct bands in the 550−1016 cm^−1^ range. The band in the wavelength range from 550 cm^−1^ to 578 cm^−1^ was associated with a triple degenerate O-P-O bending mode in PO_4_^3−^ groups which occupy two sites in the crystal lattice [46]. Characteristic of HA, the intense peaks associated with asymmetric P-O stretching vibrations were attributed to 1016 cm^−1^ while the band observed at 845 cm^−1^ corresponds to the presence of carbonate ions [47,48].

Analysis of the spectra of the composites clearly showed the peaks coming from the PVP. This was particularly noticeable for the peak at 1645 cm^−1^ attributed to the C=O bond [49]. At 1418 cm^−1^, C-H bending was observed and 1265 cm^−1^ was attributed to CH_2_ wagging [50]. It is worth noting that the peak for C-H bending was also observed in GE and SA, which was due to the chemical similarity of these polymers. Composites 17.2 and 25.2 clearly showed a signal from HA at 550 cm^−1^. However, the other signal expected to be seen in the composites, corresponding to asymmetric P-O stretching vibrations overlapped with the signal obtained from SA. This peak was attributed to the elongation of C-O groups occurring in SA [51].

### 3.3. Incubation In Vitro 

#### 3.3.1. pH-Metric Analysis

**Figure 3:**
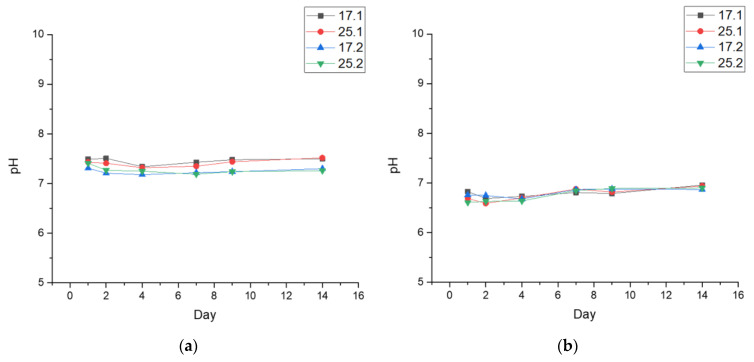

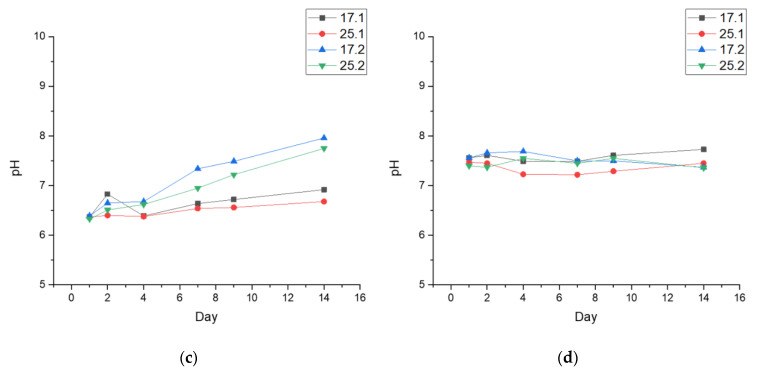
Measured pH values of: (**a**) PBS; (**b**) Ringer’s fluid; (**c**) artificial saliva; (**d**) distilled water.

Incubation studies were conducted to determine what would happen to the synthesized composites when immersed in fluids similar in composition to those found in the human body. According to the results of the study (Figure 3), it was possible to observe the curves showing the pH changes for all the tested composite samples subjected to incubation experiments. The tested materials were incubated in four different fluids, namely distilled water, PBS, Ringer’s fluid and artificial saliva. It can be clearly observed that the tested composites had an effect on the pH of the tested fluids.

The greatest changes in pH values were noticed for the samples immersed in the artificial saliva solution. There, partial degradation was also observed. Probably, this is related to the slightly acidic reaction of the fluid, whose initial value was 5.5. Therefore, it could cause the degradation process of the samples, and the substances as well as ions released from it during this process significantly influenced the pH change. Moreover, it could also be the result of partial elution of HA from the interior of the sample, as this inorganic compound is poorly soluble in such solutions and as a result the solution becomes slightly basic [52]. In the case of distilled water, there were no free ions in the incubation medium that could interact with the test samples and thus significantly affect the pH. Therefore, the observed pH changes were relatively small. Additionally, during incubation of the samples in the other fluids, i.e., PBS and Ringer’s fluid, no rapid changes were observed, and the pH value remained very similar despite the rich chemical composition of the incubation media. This is probably related to the formation of new apatite layers on the surface resulting from the presence of calcium ions in the fluids. This may limit the degradation process and thus the rapid changes in pH value. In addition, this observation may be due to the buffering properties of the composites, which lie in their ability to maintain the pH of the solution at the same level [53]. Such observations may indicate that the composites analyzed can be considered biocompatible with the selected fluids.

#### 3.3.2. Determination of Sorption Capacity of Biomaterials

**Figure 4:**
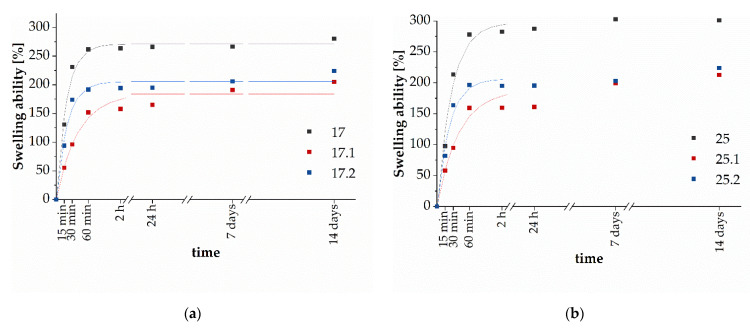
Kinetics of swelling of samples 17, 17.1, and 17.2 (**a**), and 25, 25.1, and 25.2 (**b**).

The swelling rate of hydrogels is an important parameter that controls the release patterns of solvents, drugs, and other active substances from polymer networks. The observed lower sorption capacity for composites compared to polymer matrices is probably caused by the presence of ceramics. HA powder is physically filled into the polymeric network by occupying “free spaces” in the polymer network, which can hinder the diffusion of water molecules [54]. The addition of HA has been shown to generate more crosslinking points in the polymer network, resulting in a reduction in the flexibility of the polymer chains, as well as a reduction of hydrophilic groups on the composite backbone [55]. The entire swelling process is related to the penetration of water into the matrix of the dry material and subsequent hydration of the most polar hydrophilic groups. As a consequence, the hydrogel swells, the hydrophobic groups are exposed, and they also begin to interact with water. As a result of the exposure of polar and hydrophobic sites, the polymer network has the ability to absorb additional water due to the osmotic pressure created [56,57]. The additional amount of absorbed water fills the free spaces between the polymer chains. Therefore, even small values of swelling of the material are a satisfactory result, because it is a sign of the possibility of using such a system in delivering active substances, since as a result of swelling of the hydrogel, small particles of, e.g., a drug are released. Figure 4 presents the kinetics of swelling of the tested materials. *S_e_* and *τ* values for all specimens are summarized in Table 2. 

As can be observed in Figure 4 in the initial incubation period, the swelling ability increased and then stabilized over time. The samples were characterized with different maximum swelling capacities being in the range of 205–300%. However, it is worth noticing that *Se* and *τ* values were greater for series 25. Then, it can be concluded that the addition of GE increases the swelling ability of materials, and simultaneously leads to slower liquid medium absorption. For both samples 17.1 and 25.1 containing the ceramic phase, a decrease in sorption capacity was visible. These samples demonstrated the lowest *S_e_*, but rate parameters were the highest. Therefore, it can be concluded that the addition of the ceramic phase inhibits penetration of the liquid medium into the interior of the samples. Interestingly, the presence of plant extract in the materials results in an increase in the *S_e_* values and faster liquid absorption at the initial stage of immersion in comparison with 17.1 and 17.2 samples. However, *S_e_* values are lower in comparison with unmodified samples (17 and 25). This may be caused by hydrogen bonds forming between substances contained in extract and polymers chains, which initially slowed liquid absorption, however, further incubation led to extract elution resulting in greater *S_e_* values in comparison with composites 17.1 and 25.1.

### 3.4. Determination of Total Polyphenol Content 

The total polyphenol content (TPC) was determined using the Folin−Ciocâlteu (F−C) method. This method takes advantage of the ability of polyphenols to react colorfully with Folin’s reagent. Phenolic compounds present in the sample were oxidized and the salts of phosphomolybdenum and phosphotungstic acids, which are components of the F−C reagent, were reduced in an alkaline medium—the resulting reaction product was blue in color [58]. The TPC for sage extract was expressed as gallic acid equivalent per one gram of dry weight (mg GAE, g^−1^ d.w.), and equals 16.21 ± 0.58 mg GAE·g^−1^ d.w.

### 3.5. Determination of Antioxidation by DPPH Method

The DPPH method was used to determine the antioxidant potential of common sage. According to it, 2,2-diphenyl-1-picrylhydrazyl (DPPH) radical, which is a dark purple crystalline solid, was mixed with the plant extract and the decrease in absorbance at 517 nm was measured [59]. The antioxidant capacity of common sage was presented as a percentage of inhibition, and the value reached 86.06 ± 0.49%.

### 3.6. Determination of Release Kinetics of Polyphenols

**Figure 5:**
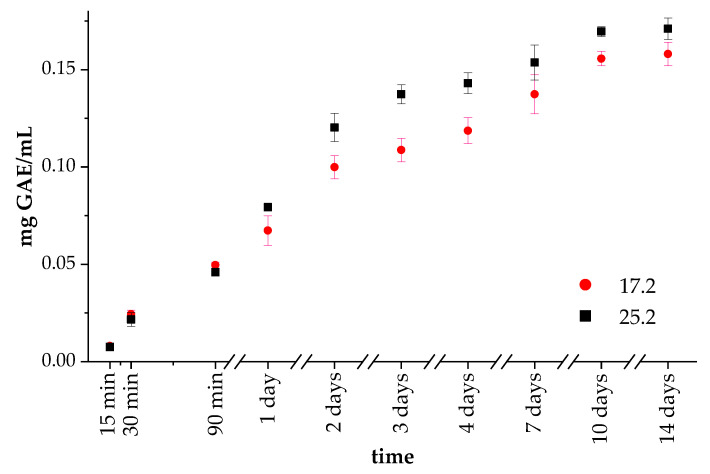
Release profiles of extract from samples 17.2 and 25.2.

Sage extract was entrapped during composite preparation, as described in Section 2.5. The preparation of the composites, and then their release behavior was examined (Figure 5). Initially, samples 17.2 and 25.2 contained the same amount of polyphenol in PVP solution. The obtained results showed the release of polyphenol at similar rates for both samples 17.2 and 25.2 during the 90 min of incubation. However, after 1 day of incubation, the TPC content in the liquid medium was greater for sample 25.2. This trend was observed during further incubation. These results are consistent with the swelling capacity measurement, which showed a higher Se value for specimen 25. The higher swelling ability of the materials led to more effective extract release. Thus, it can be concluded that the addition of GE increases the amount of extract released from materials. During the release of sage extract, it is particularly significant that the process was gradual, not rapid, or abrupt.

### 3.7. Morphology Analysis

**Figure 6:**
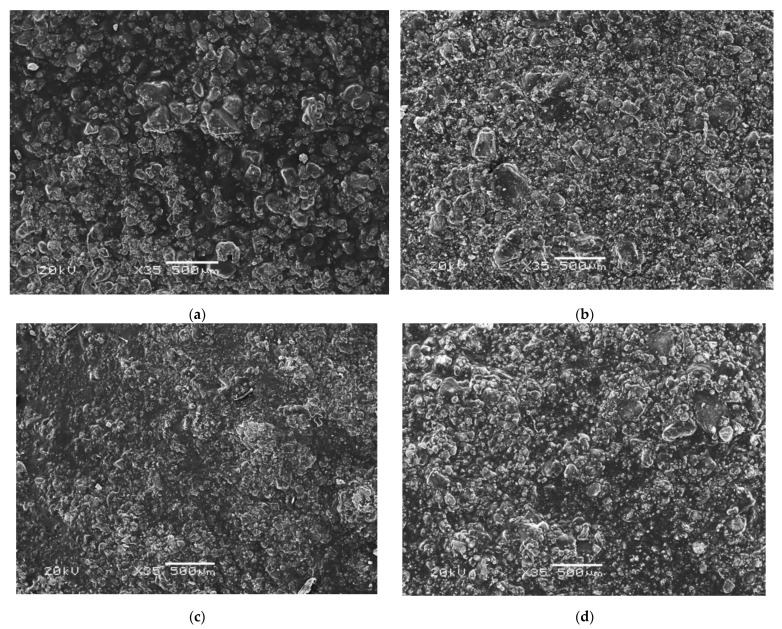
Morphology analysis of the composites (**a**) 17.2. before incubation; (**b**) 17.2 after incubation in PBS; (**c**) 25.2 before incubation; (**d**) 25.5 after incubation in PBS.

The composites were incubated for 14 days in selected artificial biological fluids. Due to the chemical composition of PBS (especially the presence of Ca and P ions) and possible interactions between PBS and the material, the most interesting results were obtained in this liquid (Figure 6). Comparing the surface of the material before and after the incubation period, it was clear that new apatite layers had appeared on their surface. The appearance of such apatite layers as well as crystals on the surface of the incubated materials is the most desirable result, as the observed biomineralization indicates the bioactivity of the composite. This confirms the interaction between the sample and the incubation fluid. Moreover, the ability to form mineralized apatite layers on the surface of incubated samples is highly dependent on the presence of HA in the biomaterial. A higher percentage of ceramic in the composite would likely result in the appearance of even more apatite layers. 

**Figure 7:**
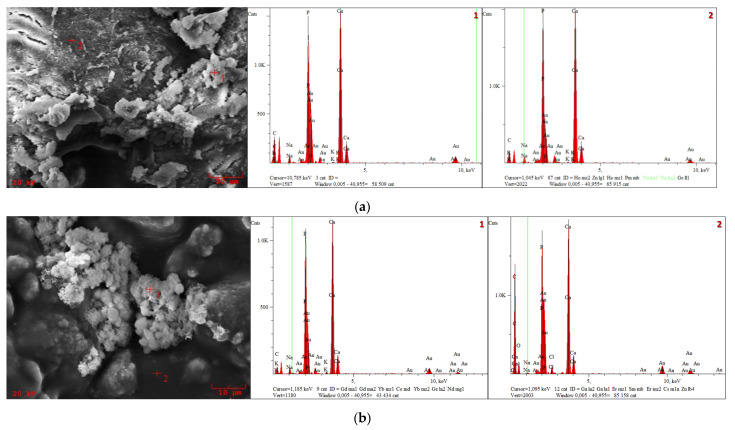
SEM morphology and EDS microanalysis of the composites: (**a**) 17.2; (**b**) 25.2.

For a more detailed analysis, EDS was additionally performed to determine the presence of individual elements (Figure 7). The presence of Au is related to the need for gold sputtering prior to SEM analysis.

Detailed EDS analysis for the presence of each element is shown in Table 3. The presence of a considerable number of C atoms was noticeable in each sample, which was closely related to the composition of the polymer matrix. The detection of K, Na and Cl ions after incubation indicates the occurrence of the desired reactions between the biomaterial and the artificial physiological fluid. In addition, analysis of the elemental composition of the composites also indicates significant amounts of Ca and P, which are the basic elements of hydroxyapatite ceramics. 

For samples incubated in artificial saliva solution, progressive degradation was observed, presumably due to the effect of the slightly acidic pH of the fluid, on the polymeric matrix. In the case of Ringer’s fluid, it seemed that blooming of new apatite crystals could be observed on the sample, however, after deeper analysis, it was determined that the polymeric matrix degrades slowly, exposing HA crystals already encapsulated in the polymeric network during the synthesis step. Nevertheless, it is worth noting that in this case, due to the slightly higher pH, the degradation process was slower than in the case of the samples immersed in artificial saliva.

### 3.8. Biological Properties of the Composites

#### 3.8.1. In Vitro Cytocompatibility of Composites

**Figure 8:**
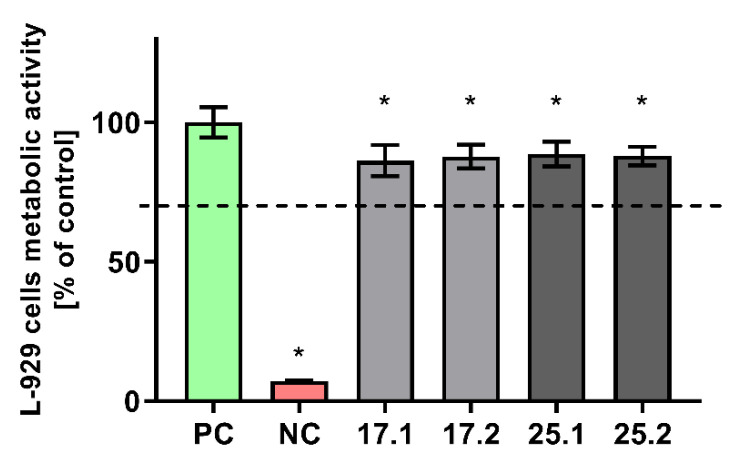
The viability of murine fibroblasts (L-929) after 24 h incubation with biocomposites, evaluated using MTT reduction test according to ISO-10993-5:2009. The positive control (PC) of the viability (100%) consisted of cells incubated without composites. The negative control (NC) of the viability (cells with significantly impaired metabolic activity) consisted of cells incubated with 0.3% hydrogen peroxide. The data are presented as mean ± SD, n = 6. The dashed line indicates the minimum level (70%) of the cells’ metabolic activity required to recognize the biomaterial as noncytotoxic at the in vitro level. * *p* values (<0.05) calculated in comparison to the NC.

The effect of polyvinylpyrrolidone/sodium alginate/hydroxyapatite composites enriched with polyphenols extracted from common sage (*Salvia officinalis*) on the metabolic activity of the mitochondrial dehydrogenases of the reference L929 murine fibroblasts was evaluated in MTT reduction assay. As shown in Figure 8, all the tested biocomposites did not affect the viability of murine L929 fibroblasts after 24 h of incubation.

The viability of murine fibroblasts exposed to 17.1, 17.2, 25.1 and 25.1 biomaterials was at the levels of 86.24% ± 5.6%; 87.77% ± 4.24%; 88.6% ± 4.44%; 87.94% ± 3.37%, respectively. A statistical analysis has shown that there was no statistical significance between the viability of cells incubated with biocomposites containing polyphenols extracted from common sage (17.2 and 25.2) and the ones that did not contain extracts (17.1 and 25.1) However, the slight decrease in the metabolic activity of L929 cells was found to be statistically significant when compared with the physiological viability of the control cells.

#### 3.8.2. Cell Colonization of Composites

**Figure 9:**
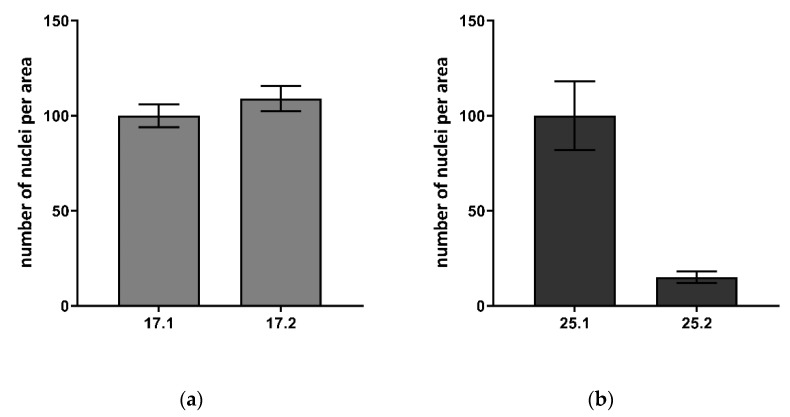
The number of nuclei per area visualized on the surface of biocomposites: (**a**) 17.1 and 17.2; (**b**) 25.1 and 25.2 after 5 days of incubation. The mean ± SD number of cell nuclei visible on at least 3 fields was counted. The 17.1 material constitutes the reference for 17.1, whereas 25.1 a reference for 25.2. The control material was set as 100%.

**Figure 10:**
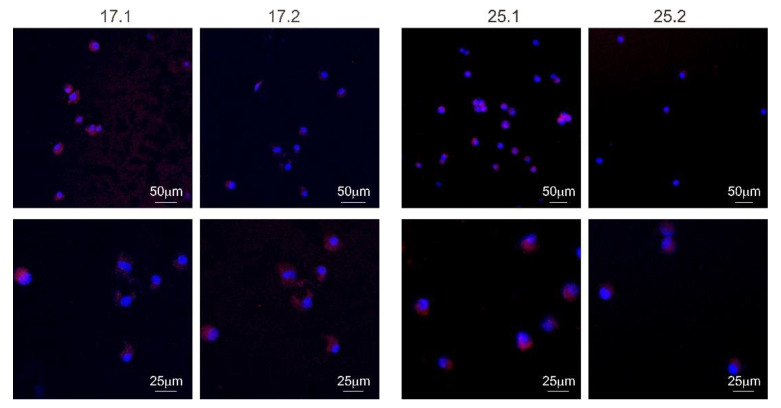
Visualization of L929 cells adhesion and expansion on the surface of biocomposites (17.1, 17.2, 25.1 and 25.2) after 5 days. The nuclei were stained with 300 nM 2-(4-amidinophenyl)-1H-indole-6-carboxamidine (DAPI) and actin filaments with phalloidin conjugated with iFluor 594. Samples were imaged with the following wavelength values of excitation and emission: 405 and 430–480 nm for DAPI, 590 and 615–630 nm for iFluor 594 conjugated antibody. Leica Application Suite X (LAS X; Leica Microsystems) was used for cell imaging.

The adhesion of L929 murine fibroblasts after 5 days of exposure to polyvinylpyrrolidone/sodium alginate/hydroxyapatite composites enriched with polyphenols extracted from common sage (*Salvia officinalis*) within their surface was investigated (Figure 9 and Figure 10).

It has been shown that fibroblasts are able to colonize the composite, however, with a different effectiveness which depends on the composition of the individual material. The number of cells identified on the biomaterial surface was as follows: 17.1–24.67 ± 6.03, 17.2–27.00 ± 6.56, 25.1–78.00 ± 14.00, and 25.2–12.00 ± 2.65. In the case of matrices 17.1 and 17.2, the number of nuclei per area was slightly higher for the composite 17.2. However, the difference was not statistically significant. In contrast, biocomposite 25.2 containing the polyphenols from common sage, was a less favorable environment for L929 cell adhesion and expansion compared to composite 25.1 lacking the extract. The addition of gelatin was important in the context of cell adhesion which is visible in the nuclei numbers in composite 17.1 (no gelatin) and 25.1 (with gelatin). No differences in the L929 cells morphology were observed between the composites containing the extract and the ones lacking the extract addition.

#### 3.8.3. Immunocompatibility of THP1-Blue NF-κB Human Monocytes with Composites

To determine the stimulatory effects of biocomposites, the activation of the NF-κB factor in monocytes was determined. THP1-Blue monocytes, as a bioindicator of the composites’ ability to induce immune cell activation, were used. Thus, because monocyte activity is crucial for the induction of a first (inflammatory) stage of the healing process, we attempted to evaluate the influence of the polyvinylpyrrolidone/sodium alginate/hydroxyapatite composites enriched with polyphenols extracted from common sage (*Salvia officinalis*) on the NF-κB induction in THP1-Blue monocytes.

**Figure 11:**
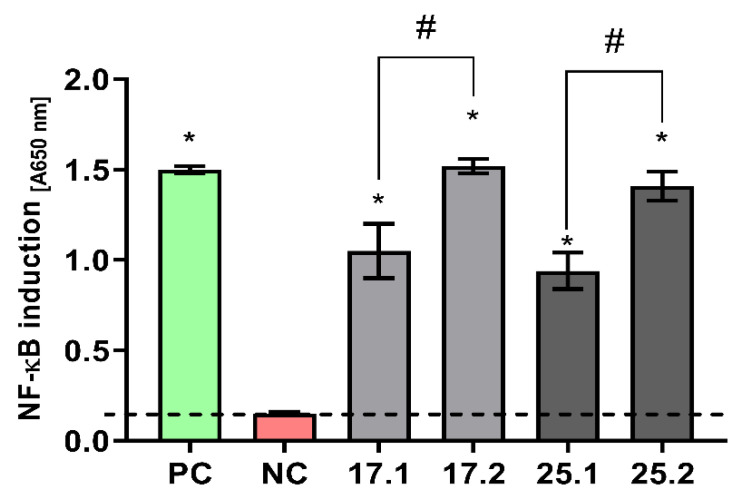
NF-κB induction in THP1-Blue monocytes incubated for 24 h with biocomposites. The negative control (NC) of the monocyte’s activation (cut-off line) consisted of cells incubated without composites. The positive control (PC) of the monocyte’s activation consisted of monocytes stimulated with PMA (100 ng/mL). The data are presented as the mean ± SD, n = 6. The dashed line indicates the physiological level (0.151 ± 0.008) of the nonstimulated monocytes. * *p* values (<0.05) calculated in comparison to the untreated cell cultures. #—statistical significance between biocomposites enriched with polyphenols extracted from common sage and their reference (17.1 vs. 17.2 and 25.1 vs. 25.2).

It has been shown that after a 24 h incubation of THP1-Blue cells in the milieu of polyphenol-enriched materials, stimulation above the cut-off value was achieved not only for the positive control with PMA but also in cultures with the tested composites. These values were statistically significant in relation to the cut-off value (unstimulated culture) (Figure 11). Moreover, the degree of stimulation in the composites enriched with polyphenols extracted from common sage (17.2 and 25.2) was statistically significantly higher than in the control matrices containing no extract (17.1 and 25.1).

## 4. Discussion

Composites based on polymers reinforced with HA and modified with common sage extract can be used in regenerative medicine of the skeletal system. The results presented indicate that polyphenols are released from the prepared composites. In this study, the XRD analysis of the synthesized hydroxyapatite showed that the material is characterized with a hexagonal structure. What is more, the analysis of the FT-IR spectrum indicated the presence of peaks and functional groups of ceramic material that showed osteoconductive properties. Comparing the obtained spectrum FT-IR of composites with pure components, one can observe the peaks coming from PVP. Furthermore, the signal from HA at 550 cm^−1^ occurs in the composite’s spectra with common sage.

To investigate the behavior of the polymer-ceramic composites in fluids simulating the human body environment, in vitro investigations were performed. The biomaterials were incubated in PBS, Ringer’s solution, artificial saliva, and distilled water. The pH-measurements of the artificial saliva solution revealed the greatest changes which were related to the partial degradation that occurred. Probably, the ions released from composites and the HA during the incubation lead to the pH increasing, causing a slightly basic solution [60]. However, no rapid changes of pH value were observed for samples immersed in PBS and Ringer’s solution. In these cases, the new apatite layers were formed on the surface. Moreover, after 14 days of incubation in distilled water, swelling capacity was lower for the polymer-ceramic composite with common sage (17.2 and 25.2) than polymer matrices (17 and 25). However, the lowest values of Se were recorded for extract-free composites (17.1 and 25.1). The liquid medium penetration into composite materials structure was also slower. The antioxidant activity and TPC of *Salvia officinalis* extract were evaluated using the Folin−Ciocâlteu method and DPPH method. TPC for common sage equals 16.21 ± 0.58 mg GAE·g^−1^ d.w., whereas the percentage of inhibition and the value reached 86.06 ± 0.49%. The evaluation of antioxidant activity as well as TPC was performed with the results by Francik et al. [35]. In accordance with the results shown, these values were dependent on the harvest period and drying method of the plant. The values of antioxidant capacity and TPC of the extract obtained with hydrochloric acid in 80% methanol were in the range 50.9 ± 9.4–60.5 ± 8.8%. and 11.6 ± 5.6.–17.1 ± 7.1 mg GAE g^−1^ d.w., respectively. The extraction from the plant was carried out by shaking at room temperature for 2 h with an Elpan shaker. Higher values of radical scavenging activity of extract obtained in our research may be caused by longer extraction time as well as the higher temperature of the process. Furthermore, the kinetics of polyphenol release from polymer-ceramic composites was examined. It proves that this process was gradual, which is crucial in the drug delivery system. Moreover, it was shown that the addition of GE resulted in a more effective extract release from materials. The addition of an aqueous extract of common sage increased the biological value of the obtained composites.

The SEM analysis of the composites in selected artificial biological fluids showed changes in morphology before and after incubation. Newly formed apatite layers could be observed in PBS, which is a very desirable effect due to the bioactivity of the composite. Detailed EDS analysis revealed the presence of calcium and phosphorus, which make up the hydroxyapatite ceramics. This fact has a positive effect on the osteoconductive properties of the bone cells, a significant factor in orthopedic applications. In the case of samples incubated in an artificial saliva solution, degradation was observed. This is due to the slightly acidic pH of the incubation fluid. During the degradation process of the polymer matrix in Ringer’s fluid, hydroxyapatite crystals were exposed, and the process itself was slower than in artificial saliva. Furthermore, the surface roughness analysis showed that the tested biomaterial samples were not perfectly flat. 

The performed analysis of the cytocompatibility of composites gave satisfactory results and confirmed the safety and application potential of the presented materials, as biomaterials are considered safe at the in vitro level in the absence of cytotoxic activity mediated by them. In this study, we have shown that polyvinylpyrrolidone/sodium alginate/hydroxyapatite composites met the ISO 10993-5:2009 criterion for maintaining the in vitro viability of at least 70% of the murine fibroblast cells exposed to the biomaterial. L929 cells are the cell type that participates in the early stages of the wound healing process. Moreover, they are able to easily adhere and proliferate on a variety of biomaterial surfaces [61]. The murine fibroblast L929 cell line was used to investigate cell adhesion and morphology on the surface of the composites.

It was apparent that the morphology of L929 mouse fibroblast cells was similar on the composites with the sage extracts to the ones without extracts. There was no evidence that sage extract increased the adhesion and proliferation of L929 cells. In the case of the tested composites, the addition of gelatin significantly increased the number of cells adhering to the surface of composite (17.1 vs. 25.1). Thus, the polyvinylpyrrolidone/sodium alginate/hydroxyapatite composites enriched with polyphenols extracted from common sage, can be considered as composites to be used as a substrate to support the adhesion and proliferation of L929 mouse fibroblasts and should be further tested. Most organ and tissue damage resulting from congenital defects, trauma or chronic disease is treated surgically or pharmacologically [1,2]. As has been mentioned, great emphasis has been placed on intelligent, bioactive biomaterials which offer great potential for regenerative medicine, by, among other properties, stimulating surrounding tissues or delivering active components such as drugs or biomolecules [3]. Thus, apart from the lack of cytotoxicity, newly synthesized composites should exhibit features promoting the healing process. The first immune cells that emerge at the site of trauma and are involved in the healing process and tissue rearrangement are monocytes. They regulate immune-tissue homeostasis due to the secretion of cytokines and chemokines [62]. The role and the activity of monocytes in postimplantation processes is essential for the clearance of damaged cells and disintegrated tissues from the site of the trauma. The biological modification of biocomposites can facilitate the transition of monocytes into the active state, which in turn will facilitate the postimplantation processes [63].

The immunomodulatory properties of sage polysaccharides have been described by Cape et al. and Ghorbani and Esmaeilizadeh [34,64]. The evaluation of the activation of THP1-Blue monocytes via the NF-κB transcription factor after incubation with polyvinylpyrrolidone/sodium alginate/hydroxyapatite composites enriched with polyphenols proved that common sage extracts are able to activate monocytes in a statistically significant way compared to composites lacking the extracts. Thus, composites with common sage extracts may promote the regeneration process by monocyte migration and activation at the site of injury. In order to fully answer the question of whether tested biocomposite immunostimulation facilitates regeneration processes, further experiments should be conducted.

## 5. Conclusions

In our study, we successfully prepared a bioactive ceramic-polymer composite modified with common sage. Physicochemical analysis, incubation studies, as well as cytotoxicity testing of the proposed solution were performed. Considering the impressive biocompatibility of the materials used and their biodegradability, the potential utility of these biomaterials in bone tissue engineering, as well as bone regeneration, has attracted much attention, mainly due to HA, which is known for its osteoconductive properties. Moreover, the gradual release in time of the active ingredients from common sage, further enhances the biological value of the biomaterial, given the broad spectrum of action of the extract from this plant. The overall biological evaluation of the composites places them as a very promising candidate for future biomedical engineering applications. Therefore, the obtained results, considering the potential of the presented biomaterials, suggest the necessity of further research, especially in the aspect of osteogenesis.

## Figures and Tables

**Table 1:** Composite materials composition.

Sample Symbol	PVP (mL)	PVP in Sage Extract (mL)	SA (mL)	GE (mL)	PEGDA (mL)	Photoinitiator (µL)	Ceramic Content (% *w*/*v*)
17	7	-	3	-	2	50	-
17.1	7	-					5
17.2	-	7					5
25	7	-	1.5	1.5			-
25.1	7	-					5
25.2	-	7					5

**Table 2:** Rate parameter (*τ*) and equilibrium swelling (*S_e_*) values of investigated materials.

Sample	*S_e_* (%)	*τ* (Minutes)
17	271.0 ± 5.7	19.0 ± 2.1
17.1	183.9 ± 8.4	37.2 ± 8.3
17.2	205.5 ± 6.7	20.1 ± 3.3
25	279.1 ± 8.3	26.7 ± 3.5
25.1	187.8 ± 10.8	40.0 ± 8.5
25.2	216.3 ± 7.0	24.7 ± 3.7

**Table 3:** Elemental composition of composites after incubation in PBS.

Sample	Spot	Atomic Percentage (wt. %)
17.2	1	C: 64.19; Na: 1.54; P: 15.38; K: 0.12; Ca: 18.75
	2	C: 49.03; Na: 2.35; P: 20.22; K: 0.17; Ca: 28.23
25.2	1	C: 41.09; Na: 2.52; P: 25.64; K: 0.39; Ca: 30.34
	2	C: 71.26; O: 13.15; Na: 0.17; P: 6.07; Cl: 0.38; Ca: 8.961

## Data Availability

The data that support the findings of this study are contained within the article.
